# Excellent results of restricted kinematic alignment total knee arthroplasty at a minimum of 10 years of follow‐up

**DOI:** 10.1002/ksa.12452

**Published:** 2024-09-09

**Authors:** Mina W. Morcos, Gautier Beckers, Andrea Giordano Salvi, Mourad Bennani, Vincent Massé, Pascal‐André Vendittoli

**Affiliations:** ^1^ Surgery Department Hôpital Maisonneuve‐Rosemont, Montreal University Montreal Quebec Canada; ^2^ Clinique Orthopédique Duval Laval Quebec Canada; ^3^ Personalized Arthroplasty Society Atlanta Georgia USA

**Keywords:** arthroplasty, kinematic alignment, knee, mechanical alignment, personalized surgery, restricted

## Abstract

**Purpose:**

While restricted kinematic alignment (rKA) total knee arthroplasty (TKA) with cemented implants has been shown to provide a similar survivorship rate to mechanical alignment (MA) in the short term, no studies have reported on the long‐term survivorship and function.

**Methods:**

One hundred four consecutive cemented cruciate retaining TKAs implanted using computer navigation and following the rKA principles proposed by Vendittoli were reviewed at a minimum of 10 years after surgery. Implant revisions, reoperations and clinical outcomes were assessed using knee injury and osteoarthritis outcome score (KOOS), forgotten joint score (FJS), patients' satisfaction and joint perception questionnaires. Radiographs were analyzed to identify signs of osteolysis and implant loosening.

**Results:**

Implant survivorship was 99.0% at a mean follow‐up of 11.3 years (range: 10.3–12.9) with one early revision for instability. Patients perceived their TKA as natural or artificial without limitation in 50.0% of cases, and 95.3% were satisfied or very satisfied with their TKA. The mean FJS was 67.6 (range: 0–100). The mean KOOS were as follows: pain 84.7 (range: 38–100), symptoms 85.5 (range: 46–100), function in daily activities 82.6 (range: 40–100), function in sport and recreation 35.2 (range: 0–100) and quality of life 79.1 (range: 0–100). No radiological evidence of implant aseptic loosening or osteolysis was identified.

**Conclusion:**

Cemented TKA implanted with the rKA alignment protocol demonstrated excellent long‐term implant survivorship and is a safe alternative to MA to improve patient function and satisfaction.

**Level of Evidence:**

Level IV, continuous case series with no comparison group.

**Abbreviations**
FJS: forgotten joint score
HKA: hip knee ankle
KA: kinematic alignment
KOOS: knee injury and osteoarthritis outcome score
KSS: knee society score
MA: mechanical alignment
PROM: patient‐reported outcome measure
RCT: randomized clinical trial
rKA: restricted kinematic alignment
SD: standard deviation from the mean
TKA: total knee arthroplasty

## INTRODUCTION

A personalized approach to total knee arthroplasty (TKA) has been proposed as an alternative to mechanical alignment (MA) to improve patient outcomes [1, 2, 9, 31]. Despite the need for more high‐quality data to recommend one technique over the other, personalized approaches have attracted great interest in recent years [35]. The pioneer in the field, Stephen Howell, proposed resurfacing the knee joint (kinematic alignment, KA), aiming to replicate the patient's native joint surface orientations and lower limb alignment [23]. Different alignments have since been suggested and are currently being investigated [18, 27, 32]. Keeping in mind (1) the wide human anatomical variations, (2) the limits of actual implant material wear resistances and (3) the current implant fixation methods [39], Vendittoli proposed the restricted KA (rKA) in 2011 [2, 7, 40]. rKA, as KA, aims to resurface the knee joint and restore the individual pre‐arthritic knee's kinematic axes and native ligament laxities but includes alignment boundaries to avoid reproducing deviant anatomies [40]. Multiple studies have shown the non‐inferiority of rKA over MA regarding patient's gait, satisfaction, forgotten joint score (FJS) and knee society score (KSS) [2, 6, 10, 12].

Even though personalized TKA alignment techniques have been promising in the short term, concerns persist as to whether the deviation from the MA‐neutral lower limb alignment would be detrimental to long‐term implant survivorship [6, 19, 33]. Only two published long‐term results of KA TKA were reported. The first study was published by Howell et al. in a cohort of 222 cemented TKAs. His implant survivorship was 97.5% for revision for any reason at 10‐years of follow‐up [16] and 93% at 16 years of follow‐up [15], matching the best MA TKA results. The second is a randomized clinical trial (RCT) comparing KA TKA to MA TKA by Dossett et al. This study reported survivorship of 81.8% in the KA group, compared to 84.1% in the MA group at a 13‐year mean follow‐up (*p* = 0.83) [11]. To determine if rKA is a valuable MA alternative and/or KA adaptation, the purpose of the present study is to report the long‐term implant survivorship, reoperation rate, clinical results measured by different patient‐reported outcome measures (PROMs) and radiographic evaluation for signs of implant dysfunction at a minimum of 10 years of follow‐up following rKA TKA.

## METHODS

**Figure 1:**
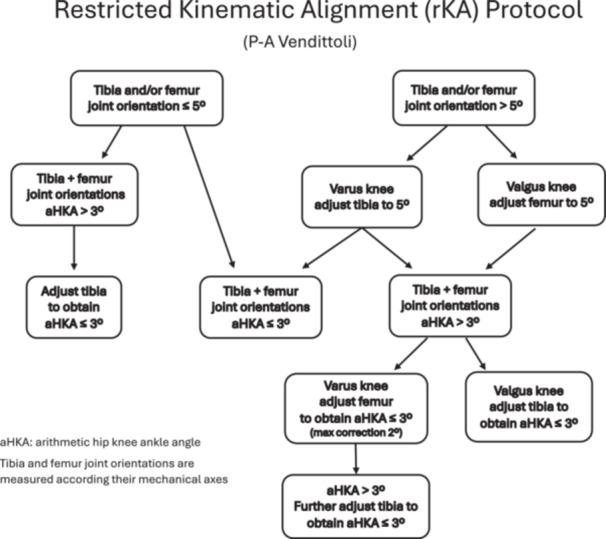
Restricted kinematic alignment protocol.

This prospective, longitudinal study followed a cohort of 104 consecutive primary rKA TKAs (89 patients), performed between April 2011 and December 2013. This cohort of patients represents the first rKA cases of the senior author at his academic centre (which represents a third of his surgical practices shared between three centres). The early results of this patient cohort have been previously published to describe the surgical technique [17]. Patients' demographics are presented in Table 1. The hospital research ethics committee approved the study, and informed consent was obtained from all the patients. All rKA TKAs were performed using a computer‐assisted navigation system (Orthomap ASM, Stryker) and received a cemented cruciate‐retaining (CR) implant (Triathlon CR, Stryker). An anterolateral skin incision and mid‐vastus approach without a tourniquet were used for all cases [20]. The surgical technique then followed the rKA algorithm in Figure 1 and the five rKA principles: (1) arithmetic HKA (hip‐knee ankle) angle should be ±3°; (2) femoral and tibial joint orientations should be ±5° from its mechanical axis; (3) native joint laxities should be restored (no gap balancing); (4) femoral anatomy preservation should be prioritized; and (5) anatomical modifications should be performed on the diseased compartment/intact compartment should be resurfaced [40]. Cartilage and bone loss thicknesses were estimated based on comparison with intact areas, and we aimed to restore the patient's pre‐arthritic alignment. For example, in a varus knee, the distal femoral and proximal tibial cut resections were, respectively, set at the implants' thicknesses for unworn cartilage surfaces of the lateral femoral condyle and tibial plateau. Then, cartilage wear thickness was assessed on the medial side bone surfaces (no wear = 0 mm, partial cartilage wear = 1 mm and subchondral bone exposed = 2 mm). The cut angle was thus adjusted to reach the desired medial resection thickness. For the tibial slope, all cuts were made at a neutral angle, as requested by the manufacturer (a 3° slope is included in the polyethylene). Resections only differed from patient anatomy when the measured angles fell outside the predefined ‘safe range’ as depicted in Figure 1, otherwise a standard KA procedure was performed. To resurface the posterior condyles, a posterior referencing guide was set to neutral rotation, thus resecting only the implant thicknesses on both posterior condyles (no femoral rotation modification). Tibial component rotation was set by its alignment with the trial femoral component with the knee in 10° of flexion. When the resection pieces did not match the computer plan or when ligament laxities assessed with trial implants were outside the expected native ligament laxity range, resection accuracy was confirmed by calliper measurements and cut adjustment was performed when needed [17, 21]. As the computer navigation system used does not allow joint gaps evaluation, knee stability was assessed by the surgeon with the trial implants, aiming to reproduce the patient's native gaps laxities (pre‐operative evaluation or intra‐operative, after osteophytes removal). This usually represented a gap of 1–2 mm medially and a gap of 2–3 mm laterally at 10° of flexion and 2 mm medially and 3–4 mm laterally at 90° of flexion. Final implants were cemented without a tourniquet.

**Table 1:** Cohort demographics.

Men : women (%)	21 : 83 (80%)
Age at surgery (mean, range; SD)	67, 33–85; 10
Age at last follow‐up (mean, range; SD)	77, 44–95; 10
Mean follow‐up (mean, range; SD)	11.3, 10–13; 1

Abbreviation: SD, standard deviation from the mean.

Implant survivorship with revision as an endpoint and reoperation for any cause were reported by patients and confirmed by medical records or imaging when available. For deceased patients, implant status at the time of death was collected from medical records or confirmed by the family. Functional assessments were performed using the knee injury and osteoarthritis outcome score (KOOS), FJS [5], patients' satisfaction and joint perception questionnaires [12]. The last follow‐up radiographic images were evaluated by two investigators blinded to the implant status and clinical scores, using the modern Knee Society Radiographic Evaluation System to assess radiolucent lines, osteolysis and signs of component loosening [26]. Moreover, the coronal alignment of the limb, femoral and tibial joint surfaces' orientations were measured on pre‐ and post‐operative long‐leg standing radiographs. The mechanical hip‐knee‐ankle angle (mHKA) corresponds to the angle subtended by the mechanical axes of the femur and tibia. It was expressed in degrees, with a negative value for varus alignment and a positive value for valgus alignment. The arithmetic HKA (aHKA) was defined as the sum of the femoral and tibial mechanical joint orientations, with a negative value for varus alignment and a positive value for valgus alignment. Finally, the surgical protocols were reviewed to assess the need for soft tissue releases.

### Statistical analyses

Continuous variables are presented as mean (minimum–maximum; standard deviation [SD]), and categorical variables as frequency and percentage. Continuous data were tested for normality using the Kolmogorov–Smirnov test. Pre‐operative and post‐operative paired categorical data were compared using the Wilcoxon signed‐rank test, whereas pre‐operative and post‐operative paired continuous data were compared using the Student's *t* test. Statistical significance was set at *p* < 0.05. All analyses were performed on SPSS 29.0 (SPSS Inc.).

## RESULTS

**Figure 2:**
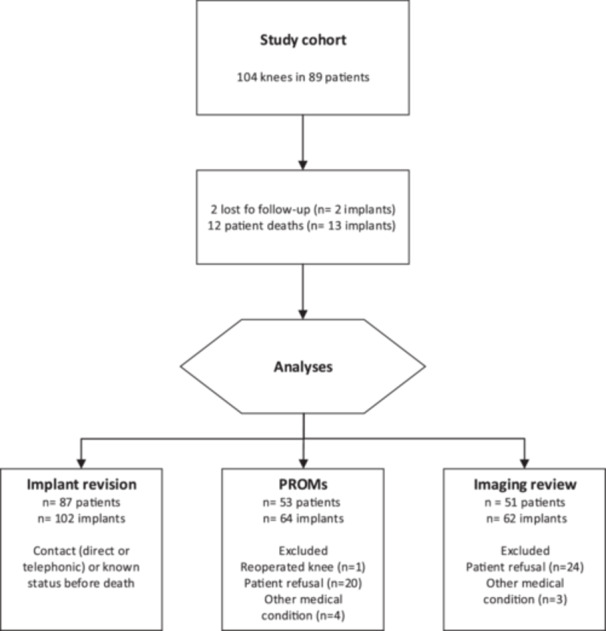
Flow chart at last follow‐up. PROM, patient‐reported outcome measure.

Of the initial cohort of 89 patients (104 knees), 12 patients (13 knees, 12.5%) died due to conditions unrelated to the surgery, and 2 patients (2 knees, 1.9%) were lost to follow‐up. We confirmed that deceased patients did not have revision surgery at the time of death, leaving 102 implants in 87 patients included in the implant survivorship analysis (study flow chart, Figure 2).

### Implant survivorship

**Figure 3:**
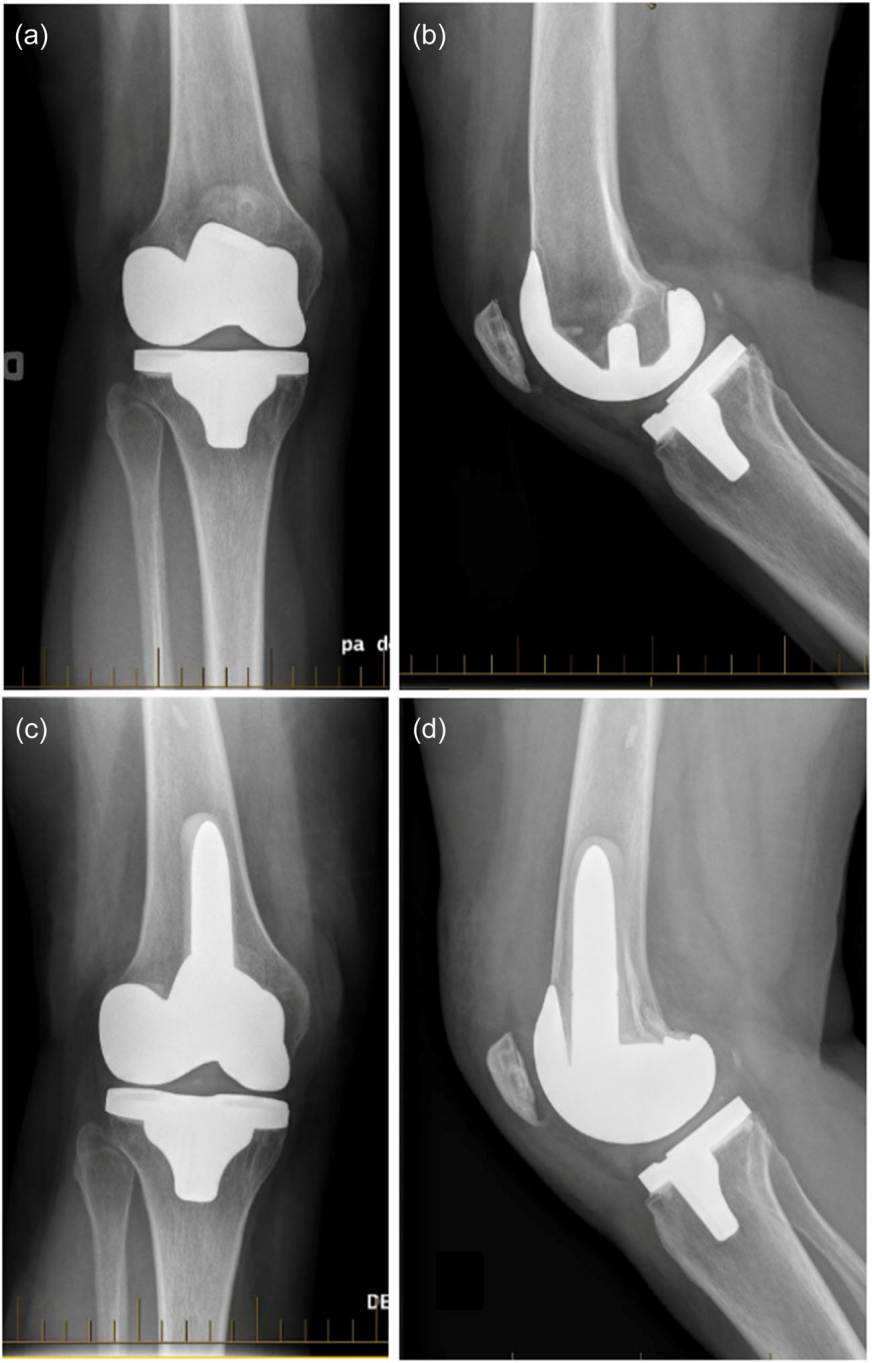
TKA instability and recurrent hemarthroses secondary to femoral component malrotation. Anteroposterior (a) and lateral (b) radiographs of the TKA before implant revision (femur 3° valgus, tibia 1° varus, HKA 2° valgus). Anteroposterior (c) and lateral (d) radiographs after revision TKA with posteromedial femoral augment and stem (femur 4° valgus, tibia 1° varus, HKA 3° valgus). Her knee phenotype did not change over the treatment process: VAL_FMA_3°/VAL_TMA_3°. HKA, hip knee ankle; TKA, total knee arthroplasty.

Implant survivorship was 99.0%. There was one early revision (1.0%) at 1‐year follow‐up for TKA instability and recurrent hemarthroses secondary to femoral component external malrotation. The patient underwent femoral component revision with posteromedial femoral augmentation and a complete synovectomy (Figure 3). Since the revision, no further hemarthrosis has occurred, and she reported being satisfied with her TKA.

**Figure 4:**
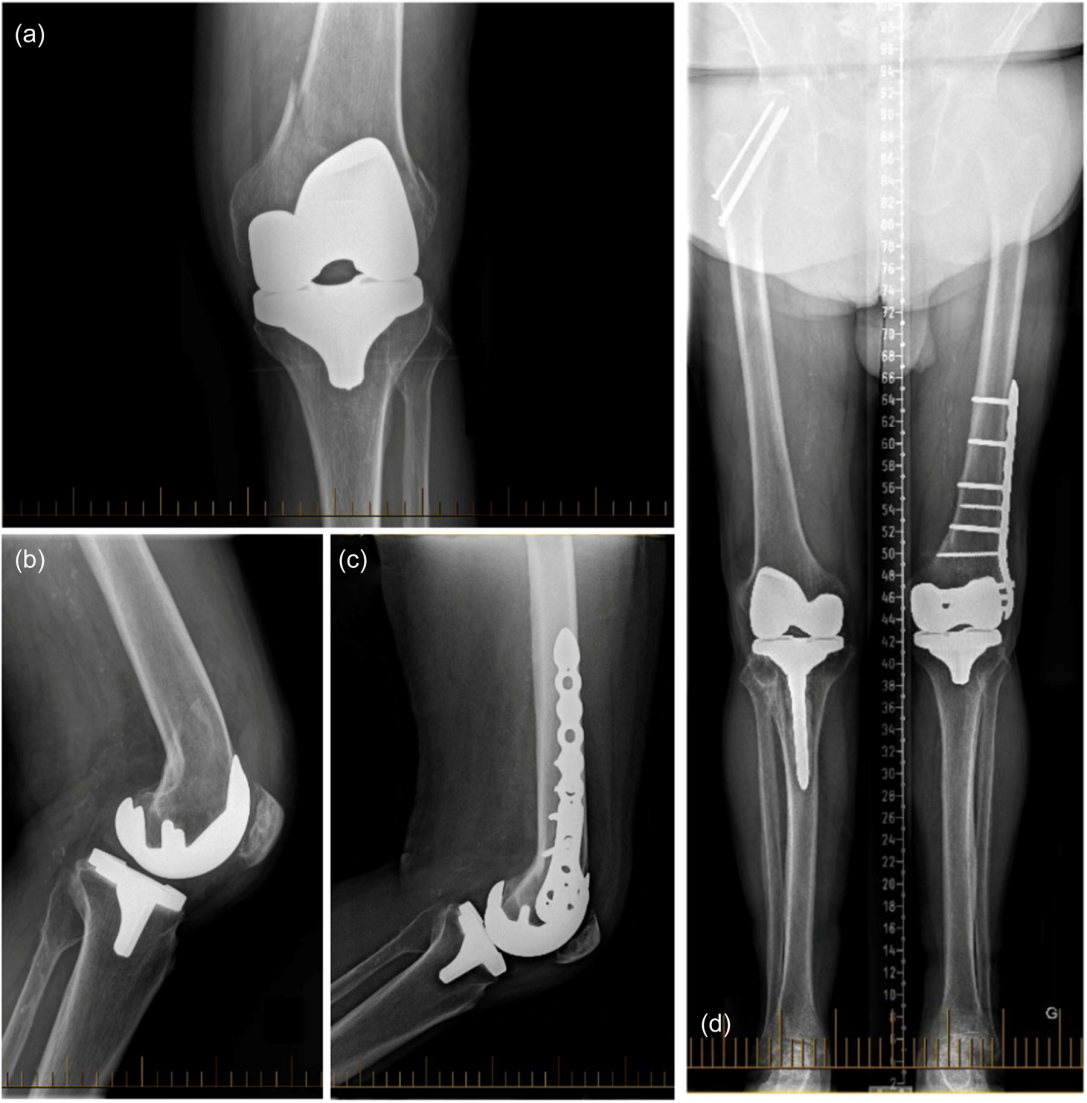
Periprosthetic TKA fracture. Anteroposterior (a) and lateral (b) radiographs of a periprosthetic femur fracture. Anteroposterior (c) and lateral (d) radiographs after open reduction and internal fixation with a lateral distal femur plate without implant replacement. TKA, total knee arthroplasty.

One patient (1%) had a femoral periprosthetic fracture following a fall from his height at the 5‐year follow‐up. The fracture was treated by femoral open reduction internal fixation without implant revision (Figure 4). Another one sustained a traumatic posterior cruciate ligament rupture following a fall at the 7‐year follow‐up. The patient was minimally symptomatic and refused surgical treatment.

### Patients' satisfaction and PROMs

Global satisfaction with surgery was 95.3% and reported as very good, good, fair and poor in 42 (65.6%), 19 (29.7%), 1 (1.6%) and 2 (3.1%) of cases, respectively. Patients reported perception of their TKA as natural, artificial without limitation, artificial with minimal limitations and artificial with major limitations in 24 (37.5%), 8 (12.5%), 26 (40.6%) and 6 (9.4%) of cases, respectively. Interestingly, of the 6 patients reporting perception as artificial with important limitations, only 2 reported poor satisfaction with surgery.

**Figure 5:**
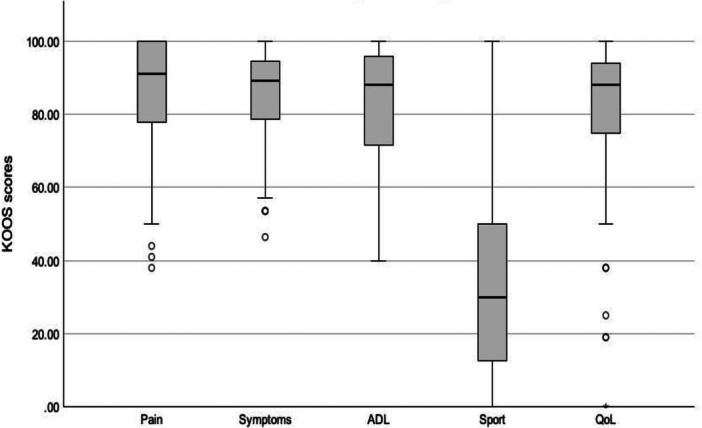
Box plot of KOOS at minimum 10 years of follow‐up. KOOS, knee injury and osteoarthritis outcome score.

The KOOS results are shown in Figure 5. The FJS mean and median were 67.6 (0–100; 28.7) and 72.9 (0–100; interquartile range: 35.4), respectively. Interestingly, among the four patients who reported an FJS of 0, two reported a perception as a natural knee, one as a knee with a minor limitation, and one with a major limitation.

### Radiographic assessment

At the last follow‐up, no osteolysis, progressive radiolucent line or implant migration was observed. Table 2 shows the pre‐operative and post‐operative long‐leg standing radiographic measurements. Most outliers according to the rKA algorithm were corrected within the rKA boundaries or nearby: 13 tibia joint surfaces were in >5° varus pre‐operatively versus 1 post‐operatively (6.0 varus), and 4 distal femurs were in >5° valgus pre‐operatively versus 1 post‐operatively (7.0 valgus). For mHKA, 39 knees were in >3° varus or valgus pre‐operatively versus 10 post‐operatively: 9 in varus with a mean of 4.8° (3.5°–6° varus) and one in 3.5° of valgus. Although it nearly reaches statistical significance, neutralization of the mHKA and aHKA seems mainly secondary to a reduction in the post‐operative tibial varus (*p* = 0.058). In contrast, no significant change in the post‐operative femoral joint surface orientation was observed (*p* = 0.905). The statistically significant difference between the pre‐operative mHKA and aHKA (*p* = 0.031) reveals the presence of asymmetric joint gaps in bipedal standing positions. These joint openings were reduced in the post‐operative comparison between mHKA and aHKA (*p* = 0.899).

**Table 2:** Pre‐operative and post‐operative measures of coronal alignment.

	Pre‐operative	Post‐operative	*p* Value
	Mean (range; SD)	Mean (range; SD)	
mHKA	5.0 varus	0.4 varus	<0.001
	(22.0 varus to 15.0 valgus; 7.8)	(6.0 varus to 3.5 valgus; 3.0)	
aHKA	1.6 varus*	0.1 varus**	0.114
	(15.5 varus to 12.5 valgus; 5.4)	(3.5 varus to 3.5 valgus; 2.2)	
Femoral joint surface orientation	2.1 valgus	2.1 valgus	0.905
	(2.0 varus to 7.0 valgus; 2.0)	(2.0 varus to 7.0 valgus; 1.7)	
Tibial joint surface orientation	3.6 varus	2.3 varus	0.058
	(19.0 varus to 11.5 valgus; 4.9)	(6.0 varus to 3.0 valgus; 1.9)	

*Note*: Paired *t* test comparing mHKA to aHKA before surgery *p = 0.031 and after surgery **p = 0.899.

Abbreviations: aHKA, arithmetic hip‐knee‐ankle angle; mHKA, mechanical hip‐knee‐ankle angle; SD, standard deviation from the mean.

### Anatomy modifications and soft tissue releases

Only five knees (5%) required ligament release, four with valgus HKA and one with varus HKA. In these five knees, the patient's anatomy fell outside the rKA boundaries of either a combined coronal orientation within ±3° of neutral and/or independent femoral or tibial cuts within ±5°. Two valgus knees (2%) required lateral retinacular release to obtain satisfactory patellar tracking.

## DISCUSSION

This is the first study to report excellent long‐term implant survivorship of 99% after a minimum of 10 years of follow‐up (mean: 11.3) in a cohort of 104 cemented rKA TKAs. Moreover, we found a very high patient satisfaction rate of 95.3%.

### Implant survivorship

In the paradigm change from a systematic MA technique to more personalized surgical procedures aiming to restore individual anatomy, concerns persist regarding the capacity of current implant materials and fixation methods to duplicate the excellent long‐term implant survivorship of MA TKA [13, 38]. For the specific implant used in this study (Triathlon CR, Stryker), Scott et al. [34] and Wylde et al. [44] reported, respectively, a 10‐year implant survivorship rate of 97.9% and 95.4% in 462 and 253 consecutive MA TKAs. However, for Scott et al., the survivorship rate dropped to 93% when accounting for any reoperation, and, in particular, they reported 10 cases (2.0%) of manipulation under anaesthesia [34]. Similarly, the Australian Orthopedic Association National Joint Replacement Registry reported a 10‐year survivorship rate of 96.4% (IC 96.2–96.5%) for the cemented Triathlon CR (Stryker) [36]. The results of the current study, using rKA in 104 TKAs, are comparable to MA, with 99% survivorship after a minimum of 10 years. Our single revision case was due to femoral component malrotation (surgical error), leading to instability and recurrent hemarthroses (Figure 3). A femoral component revision solved the problem. After analyzing the radiographs at the last follow‐up, no signs of implant loosening were observed in our cohort. Our study results are reassuring and let us believe that rKA is a safe alternative to MA.

Pioneers in the field of personalized TKA, Howell et al., reported a survivorship of 98.5% in 222 cemented KA TKAs at 10 years of follow‐up [16] and 93% after 16 years [15]. Dossett et al. [11], comparing KA TKA to MA TKA in an RCT, reported survivorship rates of 82% for the KA group and 84% for the MA group at 13‐year mean follow‐up (*p* = 0.83). Interestingly, in both long‐term KA TKA studies, the authors reported patellar complications as a main cause of reoperation (1.8% for Howell et al. [15] and 11.0% for Dossett et al. [11]). Using MA implant designs and putting the femoral component anatomically with KA (more in valgus than MA) may be unfavourable for some patients with a deviant anatomy. In contrast, in the current study, no patellar issues were detected. Furthermore, not encountering patellofemoral complications in 13 years of rKA practice, the senior author believes that rKA femoral joint orientation and aHKA boundaries help prevent this complication (some deviant anatomies might not be compatible with current implants designs) [8]. When the pre‐operative patellofemoral joint is congruent, resurfacing it should maintain its integrity. However, in the presence of a pathological patellofemoral joint before TKA (patellar tilt, subluxation, etc.), surgical adaptations should be performed. We suggest for these pathological cases to use a mid vastus or subvastus approach, limit femoral valgus to 5°, downsize the femoral component, whenin between sizes (especially for shallow trochlea), lateralize the femoral implant to the lateral condylar edge, no femoral rotation, use a femoral implant with a large trochlea and low lateral ridge, remove patella osteophytes and do not resurface. This plan works for most cases, even the deviants. If specific pathology is present: patella alta, severe lateral retinaculum contracture, malunited patella fracture, etc., it should be addressed specifically (lateral approach, resurfacing the patella with a lower and medialized dome implant, etc.).

Even with the favourable long‐term KA TKA results being reported [11, 15, 16], it is our opinion that the current literature does not provide the scientific evidence to confirm the safety of unrestricted KA for every patient [41]. It should be noted that in Howell's study, only 3% (7 out of 222) of the cases had a HKA >6° (range 9° varus to 9° valgus). This selected cohort does not reflect the range of possible alignments noted in a given population, as reported in a CT‐based measure study where 16% of the pre‐operative HKA were ≥6° (ranged 24° varus to 25° valgus) [2, 11, 16]. In a stepwise transition from a systematic approach to personalized surgery, Vendittoli et al. proposed the rKA protocol. rKA advocates for a combined lower limb coronal orientation within ±3° of neutral and joint line orientation coronal alignment within ±5° of mechanical axes [40]. These boundaries have been based on the human anatomy distribution to reproduce the anatomies within approximately 2 SDs from the mean. Other restricted protocols were also proposed. The inverse KA technique (iKA, Winnock de Grave) limits HKA to 6° varus and 3° valgus, the tibial joint line orientation to 6° varus and 2° valgus and the femoral cut should be between 6° valgus and 3° varus [43]. Similarly, MacDessi et al. have proposed restrictive alignment safe zones ranging from 6° varus to 3° valgus for HKA, 6° valgus to 3° varus for the femoral resection and 3° valgus to 6° varus for the tibial resection [25]. Defining the need for boundaries or the appropriate ones will require more data on the performance of TKA in deviant anatomies [41].

### Patients' satisfaction and PROMs

Even with proven long‐term implant survivorship, MA TKA clinical results and patients' satisfaction were perceived as suboptimal with numerous studies reporting dissatisfaction rates of around 20% [3, 14, 28]. Price et al., in an editorial, state that ‘despite excellent long‐term survivorship, more work is required to enhance this procedure and development is rightly focused on increasing the proportion of patients who have successful pain relief after surgery’ [29]. One of the sources of the problem may lie in the MA ‘one size fits all’ fundamental and all the impacts of the related anatomical modifications [4]. To improve patient satisfaction, some surgeons proposed abandoning systematic MA for more personalized surgical techniques [30, 41]. As KA, rKA aims to restore the individual's pre‐arthritic or native limb alignment by resurfacing the knee joint to reproduce a more natural joint feeling but includes alignment boundaries to address anatomical deviants. Patients' satisfaction rate in this study was 95.3%. Wylde et al. [44] and Scott et al. [34], using MA with the Triathlon CR implants (Stryker), reported a similar satisfaction rate of 88% in 253 cases and 462 cases, respectively, at 10 years of follow‐up. Dossett et al. [11], in an RCT comparing MA to KA, reported a satisfaction rate of 96% in the KA group compared to 82% in the MA group (*p* = 0.16). Unfortunately, Howell et al. did not report the satisfaction rate of their cohort, preventing comparison with current study results [16].

Regarding PROMs, Waterson et al. [42], in an RCT comparing MA to KA and using the Triathlon CR TKA (Stryker), found comparable mean KOOS between groups at 1‐year follow‐up (77.7), which is similar to our mean (74.1) at minimum 10‐years of follow‐up. Scott et al. [34] reported on 462 CR Triathlon MA TKAs and found a mean FJS of 48.2 ± 33.7 after 10‐years of follow‐up. In our rKA cohort, using the same implant and after similar follow‐up, our mean FJS was 67.6 ± 28.7. Our median FJS of 72.9 was lower than Howell et al. KA cohort with a median FJS of 88 [16]. Unfortunately, as we did not identify the cases where the native anatomy was corrected during surgery (outside the rKA boundaries), we cannot compare their FJS to the ones where a KA procedure was performed. Overall, KA and rKA patients' satisfaction rates look more favourable than MA. Regarding PROMs, the data are more conflicting, but meta‐analyses reported at least equal results [37].

### Radiographic evaluation

Using computer navigation, we reduced the 17 (8%) native tibial and femoral joint surface orientations with anatomical values outside the rKA boundary of 5° to 2 cases (1 varus at 6° and 1 valgus at 7°). None of the two persisting deviant cases were planned and are probably the results of surgical imprecisions. It must be acknowledged that the precision of computer navigation is, in the best‐case scenario, less than 1 mm or 1° [24]. Second, any tilt of the saw blade could have modified the cutting jig position, jeopardizing the system's accuracy even more. Third, implant cementation may modify the navigation target. Finally, even if the coronal knee alignment evaluation on long‐leg standing radiographs is considered reliable, the presence of sagittal or rotational deformity, genuine or perceived, can affect the accuracy of this methodology [22]. Regarding the HKA, our independent femoral and tibial cuts result in a mean aHKA of 0.1 varus with a range of 3.5 varus to 3.5 valgus. However, 10 cases had post‐operative mHKA >3°, 9 in varus with a mean of 4.8° (3.5°–6° varus) and 1 in 3.5° of valgus. Although not statistically significant, the difference between aHKA and mHKA (0.1° vs. 0.4°, 0.899) represents a joint gap imbalance in the standing bipedal radiograph observed in some cases. Following the fourth rKA principle, femoral anatomy preservation should be prioritized; the pre‐operative to post‐operative HKA modification was mainly secondary to a tibial varus reduction (from 3.6° to 2.3°, *p* = 0.058). In contrast, no significant change in the post‐operative femoral joint surface orientation was produced (*p* = 0.905).

### Study limitations

The number of patients who completed the PROMs or radiographic evaluation at the last follow‐up was affected by the 13.5% death rate (causes unrelated to the TKA). Additionally, other medical conditions, as well as refusal to come for an in‐person visit, reduced even further the number of patients available for PROMs (64 knees, 62%) and radiographic evaluation (62 knees, 60%). However, regarding our primary outcome, implant survivorship, at the last follow‐up, the information is missing only for 2% of the cases. The results of the current study should be taken with caution, as they represent the first 104 cases of rKA by a single surgeon using a specific implant and computer navigation system; thus, the results may not be generalizable. Moreover, the limited number of cases would only allow the detection of a significant statistical difference of 10% in a comparative study with MA cases (with an alpha error of 5% and a power of 80%). However, the patients in this study represent a continuous series of cases performed in an academic centre using very few exclusion criteria. Therefore, we expect them to correspond to the general spectrum of patients needing TKA.

Overall, this study's results support the clinical values and safety of the rKA protocol proposed by Vendittoli. As most cases were within the rKA boundaries, anatomical modifications were performed only for deviant cases (8% of tibias and femurs were outside rKA boundaries). Related soft tissue releases to restore ligament balance were required in only five cases (5%). Avoiding reproducing deviant anatomies might be protective for some specific KA complications like patellofemoral problems. As a stepwise transition from MA to a more personalized procedure, rKA represents a valuable and safe alternative to MA. According to implant material and fixation method performances, future research should define the real boundaries if there are some needed. Furthermore, apart from the frontal plane measurements, the decisional algorithm should include a sagittal and axial plane analysis.

## CONCLUSION

After a minimum follow‐up of 10 years, rKA TKAs provide excellent implant survivorship of 99% and a high patient satisfaction rate of 95%. This suggests that restoring patients' natural alignment, respecting boundaries and avoiding deviants may be a safe alternative to MA to improve patient satisfaction. However, further RCT long‐term studies are needed to support our findings.

## AUTHOR CONTRIBUTIONS

Mina W. Morcos: Acquiring, analyzing and interpreting the data, Drafting and Critically revising the manuscript. Gautier Beckers: Acquiring, analyzing and interpreting the data, drafting and critically revising the manuscript. Andrea Giordano Salvi: Data collection, critically revising the manuscript. Mourad Bennani: Data collection, critically revising the manuscript. Vincent Massé: Analyzing and interpreting the data, critically revising the manuscript. Pascal‐André Vendittoli: Designing the study, data collection, analyzing and interpreting the data, drafting and critically revising the manuscript. Data transparency. The authors declare that all data materials and software applications support their published claims and comply with field standards.

## CONFLICT OF INTEREST STATEMENT

The authors declare no conflict of interest.

## ETHICS STATEMENT

The hospital research ethics committee approved the study, and informed consent was obtained from all the patients.
